# CBF oscillations induced by trigeminal nerve stimulation protect the pericontusional penumbra in traumatic brain injury complicated by hemorrhagic shock

**DOI:** 10.1038/s41598-021-99234-8

**Published:** 2021-10-04

**Authors:** Chunyan Li, Kevin A. Shah, Keren Powell, Yi-Chen Wu, Wayne Chaung, Anup N. Sonti, Timothy G. White, Mohini Doobay, Weng-Lang Yang, Ping Wang, Lance B. Becker, Raj K. Narayan

**Affiliations:** 1 Translational Brain Research Laboratory, The Feinstein Institutes for Medical Research, 350 Community Drive, Manhasset, NY 11030 USA; 2 Department of Neurosurgery, Zucker School of Medicine at Hofstra/Northwell, Hempstead, NY USA; 3 Center for Immunology and Inflammation, The Feinstein Institutes for Medical Research, Manhasset, NY USA; 4 Department of Radiation Oncology, Albert Einstein College of Medicine, Bronx, NY USA; 5 Department of Emergency Medicine, Zucker School of Medicine at Hofstra/Northwell, Hempstead, NY USA

**Keywords:** Nervous system, Trauma, Preclinical research

## Abstract

Traumatic peri-contusional penumbra represents crucial targets for therapeutic interventions after traumatic brain injury (TBI). Current resuscitative approaches may not adequately alleviate impaired cerebral microcirculation and, hence, compromise oxygen delivery to peri-contusional areas. Low-frequency oscillations in cerebral blood flow (CBF) may improve cerebral oxygenation in the setting of oxygen deprivation. However, no method has been reported to induce controllable oscillations in CBF and it hasn’t been applied as a therapeutic strategy. Electrical stimulation of the trigeminal nerve (TNS) plays a pivotal role in modulating cerebrovascular tone and cerebral perfusion. We hypothesized that TNS can modulate CBF at the targeted frequency band via the trigemino-cerebrovascular network, and TNS-induced CBF oscillations would improve cerebral oxygenation in peri-contusional areas. In a rat model of TBI complicated by hemorrhagic shock, TNS-induced CBF oscillations conferred significant preservation of peri-contusional tissues leading to reduced lesion volume, attenuated hypoxic injury and neuroinflammation, increased eNOS expression, improved neurological recovery and better 10-day survival rate, despite not significantly increasing CBF as compared with those in immediate and delayed resuscitation animals. Our findings indicate that low-frequency CBF oscillations enhance cerebral oxygenation in peri-contusional areas, and play a more significant protective role than improvements in non-oscillatory cerebral perfusion or volume expansion alone.

## Introduction

Clinical outcomes after traumatic brain injury (TBI) are significantly worsened by concomitant hemorrhagic shock (HS) due to increased reduction of cerebral blood flow (CBF) leading to hypoxia, approximately doubling the mortality rate compared with TBI alone^1–3^. Despite advances in the management of TBI complicated by HS (TBI + HS), the current resuscitative approach to TBI + HS still entails immediate intravascular volume expansion, vasopressors, or oxygen supplementation^4–6^. However, these interventions do not adequately alleviate impaired cerebral microcirculation in peri-contusional areas following injury^7–9^, and hence provide limited neuroprotection and improving neurological outcomes. Given these challenges, an optimal resuscitative approach to TBI + HS remains elusive.

A major consequence of TBI is direct damage to the cerebral vasculature, leading to hemorrhage, CBF abnormalities, blood–brain barrier (BBB) disruption, and edema^10–12^. These early events are followed by hypoperfusion and compromise of the cerebral microvasculature resulting in capillary stasis, ischemia and hypoxic tissue damage. All of these detrimental effects are exacerbated by HS. In TBI, in addition to hypoperfusion, endothelial swelling and perivascular edema occur, which require oxygen to travel a longer distance before reaching the injured mitochondria^13,14^. Therefore, neuroprotective strategies targeting the cerebral microcirculatory bed in order to enhance cerebral microcirculation and, subsequently improve oxygen delivery to the injured brain tissue, are highly required.

Several studies, from clinical findings to computational models, indicate significant effect of low-frequency oscillatory patterns of CBF to maintain tissue perfusion in the setting of oxygen deprivation^15,16^. While the precise mechanism of these oscillatory patterns is not well understood, it might be linked to multiple protective mechanisms for compromised cerebrovascular regulation and cerebral oxygenation. Studies in a human model of lower body negative pressure (LBNP) have shown that spontaneous low-frequency oscillations (0.04–0.15 Hz) in CBF are protective in a central hypovolemic state^17–20^. These studies suggest that such oscillations may represent an “on–off” feedback mechanism and provide a pump-like effect leading to improved tissue perfusion via temporary increases in the pressure gradient down the vascular tree. This hypothesis has been further supported by computational simulation, wherein low-frequency CBF oscillations supply higher amplitudes of localized oxygen delivery relative to oscillations at a higher frequency^16^. Furthermore, pulsatile blood flow can increase shear stress on the vessel endothelium which stimulates the release of nitric oxide and inhibits endothelin production, thus increasing oxygen delivery to brain tissue^18,21^. In this study, we therefore hypothesize that artificially induced low-frequency oscillation in CBF could improve cerebral perfusion and oxygenation in areas of brain that are damaged but not yet dead, and thereby improve overall functional outcomes following TBI + HS. However, based on our knowledge, no method has been reported to induce controllable oscillations in CBF.

The trigeminal nerve (CN V), the largest cranial nerve, innervates the majority of the cerebral vasculature including large arteries, arterioles, capillaries, pial vessels, and venous sinuses, as well as maintains associations with numerous vasoregulatory regions^22–24^. Hence, electrical stimulation of the trigeminal nerve (TNS) plays a pivotal role in modulating cerebrovascular tone and cerebral perfusion under normal and pathological conditions^25–28^. We, therefore, further hypothesize that TNS can modulate CBF at the targeted frequency band via its rich network of connections to enhance cerebral macro- and microcirculation. To test this hypothesis, we established a model of severe TBI + HS using rats and explored the beneficial effects of TNS-induced low-frequency oscillations on CBF, lesion volume, hypoxic stress, endothelial nitric oxide synthase (eNOS), neuroinflammation, neurological function, and 10-day survival rate.

## Methods

### Animals and ethics

All experiments were approved by the Institutional Animal Care and Use Committee of the Feinstein Institutes for Medical Research and performed in accordance with the National Institutes of Health guidelines for the use of experimental animals. Rats (315–390 g) (Taconic Biosciences, Germantown, New York) were housed in a temperature-controlled room (12 h light/dark cycle) and were allowed food and water ad libitum. Cages were lined with Enrich-o’Cobs bedding (The Andersons, Inc, Maumee, Ohio). Prior to surgery, rats were housed in groups of three per cage, while they were housed singly after the surgery. Results are reported in accordance with ARRIVE (Animal Research: Reporting of In Vivo Experiments) guidelines^29^.

### Rat model of TBI complicated by HS

In male Sprague–Dawley rats, a controlled cortical impact (CCI) model was used to induce severe TBI immediately followed by pressure-controlled blood withdrawal to induce HS. Rats were anesthetized with constant aerosol isoflurane delivered in medical air (5% isoflurane for induction in the chamber; 2.5–3% during surgery through the nose cone; 1.25–1.5% maintenance through the nose cone), and were placed in a stereotaxic frame on a heating plate to maintain body temperature at 37.0 ± 0.2 °C. A 6 mm circular craniotomy was performed halfway between bregma and lambda in the parietal bone centered at 4 mm lateral from the sagittal suture. CCI was delivered over this craniotomized portion of the skull with an electromagnetic-based device set (Impact OneTM Stereotaxic CCI Instrument, Leica Biosystems) using the following impact parameters: velocity: 6 m/s; depth of penetration: 3 mm; dwell time 100 ms^26^. Immediately after CCI, 50 ± 2.8% of the blood volume was removed while maintaining the mean arterial blood pressure at 27 ± 2 mmHg for 35 minutes^30^. At the end of the induced hemorrhage, rats were treated with TNS induced oscillations at 0.1 Hz (unless otherwise specified) or left untreated for 60 min, followed by an infusion of intravenous fluid with 2× Lactated Ringer’s solution for 60 min. After each surgery, rats were given subcutaneous buprenorphine (0.05–0.1 mg/kg) and returned to their home cages.

### Experimental groups

A total of 109 rats were entered into the study. Animals were randomly assigned to four groups: (1) Sham: received sham surgery and sham stimulation; (2) Delayed resuscitation (DR): received fluid resuscitation at 60 min after TBI + HS onset; (3) Immediate resuscitation (IR): received intravenous fluid (IVF) with 2× Lactated Ringer’s solution immediately after TBI/HS onset; (4) Low frequency oscillation (LFO): received low-frequency CBF oscillations (0.1 Hz) induced by TNS for 60 min immediately after TBI/HS onset and followed by IVF resuscitation;. IR group represents the best systemic hemodynamic and cerebral perfusion without the above-mentioned low-frequency oscillations. All groups underwent the same surgery procedures including performing craniotomy and placing electrodes for TNS, but stimulation was only applied to the LFO groups. To make sure that the rats experienced a similar shock condition, we selected the rats for analysis through a combined assessment of blood gas and metabolic variables (lactate: 8–11 mM; glucose: > 500 mg/dL; pH: 7.26–7.32, hemoglobin: 10.2–11.5 g/dL) at the TBI + HS onset. The blood was drawn from the femoral artery to measure the lactate, glucose, pH, and hemoglobin (Hgb) levels (i-STAT CG4 + and CG8 + Cartridge, Abbott Point of Care Inc., USA). Rats were sacrificed at either 5 or 24 h after induction of TBI + HS for sample collection.

### Induction of CBF oscillations by trigeminal nerve stimulation

Bilateral bipolar electrodes (concentric-bipolar 26 G EMG needle electrode, Natus Neurology Incorporated, Madison, WI) were inserted percutaneously between the inner border of the supraorbital ridges and the orbital contents to a depth of approximately 4 mm^24^. We have previously shown that electrodes inserted at this position can stimulate the infraorbital and anterior ethmoidal branches of the trigeminal nerve^26^. The stimulation parameters were calibrated to induce low-frequency oscillations of CBF in the frequency range of 0.05–0.4 Hz. Rectangular biphasic pulses were delivered with a frequency of 50 Hz, inter-pulse period of 20 ms, burst width of 5 s followed by a 5 s delay (unless otherwise specified) over 1 min by an electrical stimulator (Isolated Pulse Stimulator Model 2100, A-M Systems, Sequim, WA). We repeated this pattern every 10 min for a total stimulation period of 60 min.

### Monitoring of cerebral and physiologic parameters

Arterial blood pressure (BP) was continuously monitored and recorded for all the rats. A catheter placed in the left femoral artery was connected to a blood pressure transducer (MLT0670, ADInstruments, USA) for continuous recording of BP. Needle-type electrodes (MLA1213, ADInstruments, USA) were inserted subcutaneously to the hind and forelimbs bilaterally to continuously record the ECG. A laser Doppler probe to measure CBF was positioned over a 2 mm craniotomized portion of the frontal bone ipsilateral to the site of CCI. All data were digitized at 1 kHz with PowerLab digitizer (PowerLab 16/SP, ADInstruments, USA).

### Quantification of brain inflammatory markers

Brain samples were collected at 5 h or 24 h after TBI + HS. The left hemisphere was excised, rinsed of blood, homogenized with polytron in a homogenization buffer (phosphate-buffered saline solution, containing 0.05% Triton X-100 and a protease inhibitor cocktail; pH, 7.2; 4 °C) and solicited for 10 s. Homogenates were centrifuged at 16,000 g for 20 min. TNF-α and IL-6 were determined by using assay kits according to the manufacturer’s instructions (Abcam, USA).

### Quantitative, real-time PCR

mRNA was extracted from left hemispheres at 5 h or 24 h after TBI + HS using Trizol reagent (Life Technologies, Carlsbad, CA). High capacity cDNA Reverse Transcription Kit (Applied Biosystems, Foster City, CA) was used to synthesize cDNA from the isolated RNA. Primers for eNOS (forward, 5′-TGA GCA GCA CAA GAG TTA CAA AAT C-3′; reverse, 5′-GCC GCC AAG AGG ATA CCA-3′) and iNOS (forward, 5′-GGA GAG AGA TCCGGT TCA CAG T-3′; reverse, 5′-ACC TTC CGC ATT AGC ACA GAA-3′) were utilized. qPCR was performed on a 7500 Real-time PCR System (Applied Biosysems, Foster City, CA) utilizing SYBR Green PCR Master Mix reagents (Applied Biosystems, Foster City, CA). Rat GAPDH (forward, 5′-AGG TTG TCT CCT GTG ACT TC-3′; reverse, 5′-CTG TTG CTG TAG CCA TAT TC-3′) was used as an endogenous control. The delta-delta calculation method was utilized to obtain fold change relative to controls.

### Tissue processing and cryosectioning

At 24 h after induction of polytrauma, the animals were deeply anesthetized with isoflurane, and transcardially perfused with cold phosphate-buffered saline (PBS), followed by cold 4% paraformaldehyde (PFA) in PBS as a fixative. The brains were removed and immersed in PFA overnight, then cryopreserved in gradient sucrose solutions from 10 to 20% for 48 h, embedded in a 1:3 mixture of 30% sucrose and Optimal Cutting Temperature Compound (Thermo Fisher Scientific, Waltham, MA) and stored at − 80 °C for future cryosectioning. The brains were serially cut into 14 µm thick coronal cryosections from caudal to rostral at 400 µm intervals using a cryostat (Leica Biosystems, Germany), mounted on Superfrost Plus glass slides (Thermo Fisher Scientific, Waltham, MA) and Polysine glass slides (Thermo Fisher Scientific, Waltham, MA), and stored at − 80 °C.

### Measurement of lesion volume

To measure lesion volume, the serial brain sections were stained with hematoxylin and eosin Y (H&E), and digital images of the sections were acquired using a PathScan Enabler 5 (Meyer Instruments, Houston, TX). The lesion volume was calculated using ImageJ software (NIH, Bethesda, MD) and was expressed as the percentage of total ipsilateral and contralateral cerebral hemisphere volume.

### Immunofluorescence (IF) staining and microscopy

For IF staining analysis, Polysine slides were washed with 1× tris-buffered saline (TBS) containing 0.05% Tween-20 (TBST), blocked with 5% goat serum (Abcam, Cambridge, MA) supplemented with 1% bovine serum albumin (Sigma, St. Louis, MO) for 1 h at room temperature and sequentially incubated with primary antibody, mouse anti-HIF-1α antibody (Abcam, Cambridge, MA), at 4C overnight and its corresponding secondary antibody, Alexa Fluor 488 conjugated goat anti-mouse antibody (Invitrogen, Carlsbad, CA), at room temperature for 1 h. The slides were then co-stained with the primary antibody, mouse anti-NeuN antibody (Abcam, Cambridge, MA), and its corresponding secondary antibody, Alexa Fluor 555 conjugated goat anti-mouse, at room temperature for 1 h. The primary antibodies were diluted in blocking solution. The wash step between incubation was 5 min × 4 times using 1× TBST. The slides were counterstained with DAPI (1:1000, Thermo Fisher Scientific, Waltham, MA) and mounted with Vectashield Antifade mounting medium (Vector Laboratories, Burlingame, CA) Staining slides were visualized and imaged with EVOS M7000 imaging system (Thermo Fisher Scientific, USA) using 20× objective and automate function of XY-stitching to obtain whole brain images.

### Quantitative analysis of immunofluorescent-positive cells

ImageJ (National Institutes of Health, Bethesda, MD) was used to count the immunofluorescent positive cells. Post-processing of immunofluorescent images has been described elsewhere. In brief, for HIF-1α positive cell count, green channel overlapped with DAPI channel were manually counted in the region of interest (ROI) in dimension of 517 by 388 µm^2^. All quantitative analysis were performed by investigators blinded to the group allocation.

### Modified neurological severity scale

Prior to the collection of samples at 24 h, neurological function and behavioral damage was quantified using the previously described modified neurological severity scale (mNSS)^31^. Briefly, neurological function was graded based on motor score (0–10), which evaluated limb movement, ambulation, and reflexes; a sensory score (0–2), which evaluated response to visual and tactile stimuli and proprioception; and a balance score (0–6) which was determined by placing the rat upon a suspended wooden beam (2.0 cm × 4.0 cm × 60.0 cm) for up to 60 s. A maximum total score was18, with higher scores indicating poorer neurological function.

### Survival study

A 10-day survival study was conducted to determine if TNS-induced CBF oscillations improve survival in an animal model of TBI + HS. Following the experimental procedures described above, rats were returned to their cages and allowed food and water ad libitum. All surviving rats were sacrificed on day 10.

### Statistics

All data are expressed as mean ± standard deviation (SD) and analyzed by GraphPad Prism software. Survival analysis was performed by the Kaplan–Meier method and compared by the log-rank test. Effect sizes were quantified using Cohen's *d* for Student’s t-test. The CBF was analyzed using repeated measures ANOVA. The difference between multiple groups was analyzed by one-way ANOVA and post-hoc test. Student’s t-test was used when only two groups were compared. All groups were analyzed for normality using the Shapiro-Wilkes method. *P* values of less than 0.05 were considered significant.

## Results

### TNS can induce CBF oscillations at the target low frequency in both normal and TBI brains

**Figure 1:**
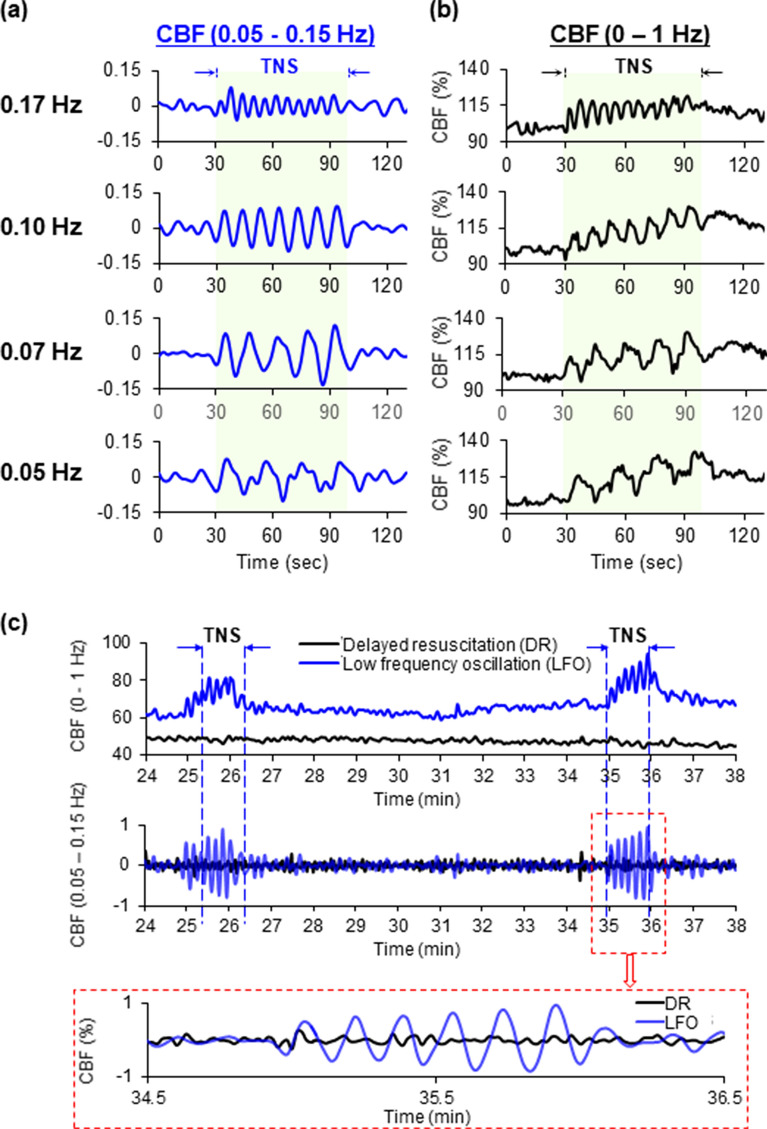
(**a**) TNS induces controllable and repeatable CBF oscillations in the target low-frequency range of 0.05–0.15 Hz. (**b**) During the stimulation, TNS gradually improves the overall peak and average amplitudes of CBF. (**c**) After TBI + HS, TNS can induce the targeted low-frequency CBF oscillations. The representative recordings of TNS-induced CBF oscillations at 0.1 Hz after TBI + HS are shown. All of the data are presented as mean ± SD. *p < 0.05 versus DR, ^#^*p* < 0.05 versus IR, n = 10 for each group. CBF: cerebral blood flow; CCI: controlled cortical impact; DR: the animals received 60 min delayed fluid resuscitation; IR: the animals received immediate fluid resuscitation; LFO: the animals received low-frequency CBF oscillations immediately after TBI + HS onset and followed by fluid resuscitation; TNS: trigeminal nerve stimulation.

The low-frequency oscillations in CBF (0.04–0.15 Hz) could be reliably generated by TNS using the stimulation parameters described in our methods. The oscillation frequency was mainly determined by stimulation duty cycle. As shown in Fig. 1, the oscillation frequencies of 0.17 Hz, 0.10 Hz, 0.07 Hz, and 0.05 Hz were generated by the duty cycle of 3-s ON/3-s OFF, 5-s ON/5-s OFF, 7.5-s ON/7.5-s OFF, and 10-s ON/10-s OFF, respectively. In normal brains, TNS not only generated controllable and repeatable CBF oscillations in the targeted low-frequency range (Fig. 1A), but also gradually improved the overall absolute peak and average amplitudes (Fig. 1B). Similarly, after TBI + HS, CBF oscillations at 0.1 Hz were induced by the duty cycle of 5-s ON/5-s OFF as shown in Fig. 1C.

### Effect of TNS-induced CBF oscillations on cerebral hemodynamics following TBI + HS

**Figure 2:**
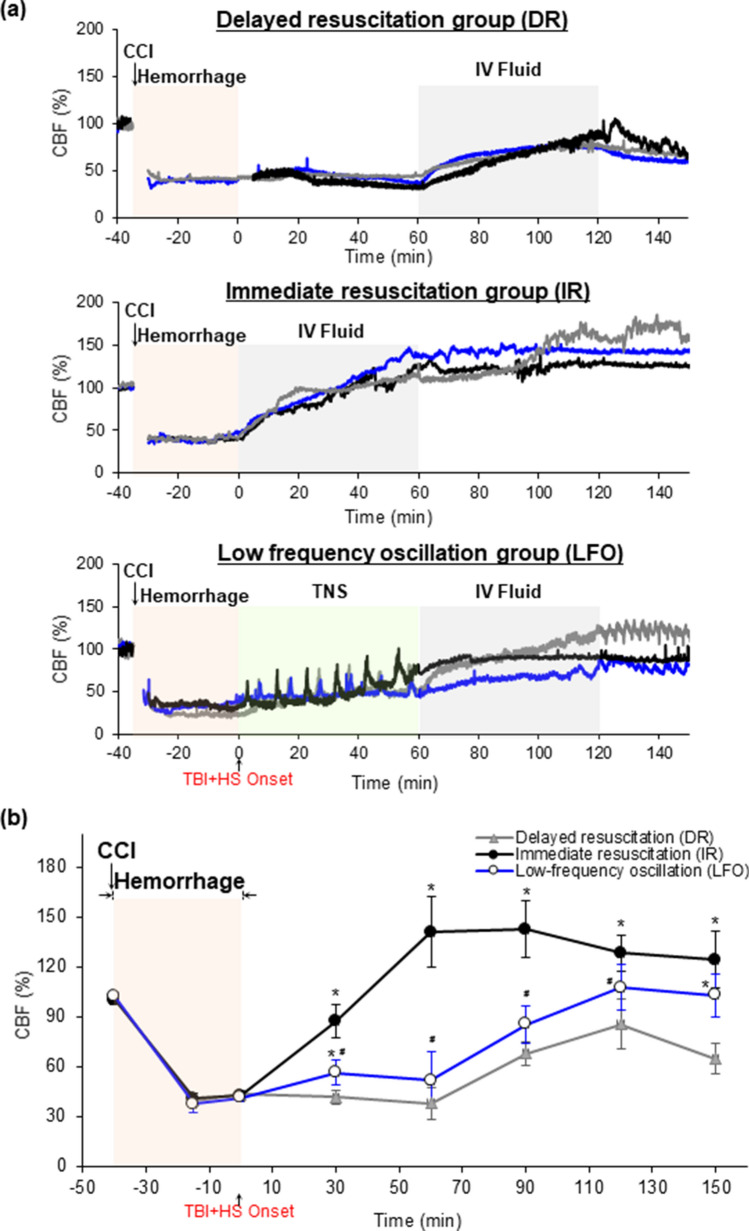
(**a**) The representative recordings of CBF from each experimental group before, during, and after TBI + HS. Pink box: during hemorrhage; Gray box: during fluid resuscitation; Green box: during TNS-induced CBF oscillations at 0.1 Hz. n = 3/group. (**b**) Effect of delayed and immediate fluid resuscitation, TNS-induced CBF oscillations on cerebral hemodynamics following TBI + HS. All of the data are presented as mean ± SD. *p < 0.05 versus DR, ^#^*p* < 0.05 versus IR, n = 10 for each group. CBF: cerebral blood flow; CCI: controlled cortical impact; IV: intravenous; TNS: trigeminal nerve stimulation.

CBF in the peri-contusional cortex was measured. TBI + HS significantly decreased the amplitude of CBF in peri-contusional brain tissues. Representative recordings in CBF for DR, IR and LFO animals are shown in Fig. 2A. At 5 min after TBI + HS onset, the TNS-induced CBF oscillations at 0.1 Hz were delivered for 1 min every 10 min. TNS generated reproducible CBF oscillations after TBI + HS, whereas no CBF oscillations were observed in either DR or IR animals in the frequency range of 0.05–0.4 Hz, as shown in Fig. 1C. The CBF amplitudes decreased to 43.4 ± 2.7% at TBI + HS onset (0 min) for all the experimental groups (Fig. 2B). Immediate fluid resuscitation significantly improved the CBF to 141 ± 21% at 60 min after TBI + HS. Without fluid resuscitation, the CBF amplitude kept decreased for DR animals to 37.9 ± 9.3% until the IVF was delivered at 60 min after TBI + HS. TNS-induced CBF oscillations didn’t significantly improve the CBF before the fluid resuscitation at 60 min, as compared with those in DR animals (37.9 ± 9.3% vs. 51.9 ± 13.8%; DR vs. LFO; Cohen’s d = 1.04). Fluid resuscitation significantly improved the CBF both in DR and LFO animals. However, after withdrawal of fluid resuscitation at 120 min, the DR animals demonstrated 20% decrease in CBF at 150 min, whereas the animals treated with the TNS-induced CBF oscillations maintained elevations in CBF amplitude (64.7 ± 9.1% vs. 102.8 ± 13.6%; DR vs. LFO; Cohen’s d = 3.41). These observations demonstrate that the TNS-induced low-frequency CBF oscillations did not significantly improve the CBF as compared to the IR animals, however, it did preserve cerebral perfusion in peri-contusional brain tissues.

### Induced low-frequency oscillations in CBF reduced lesion volume

**Figure 3:**
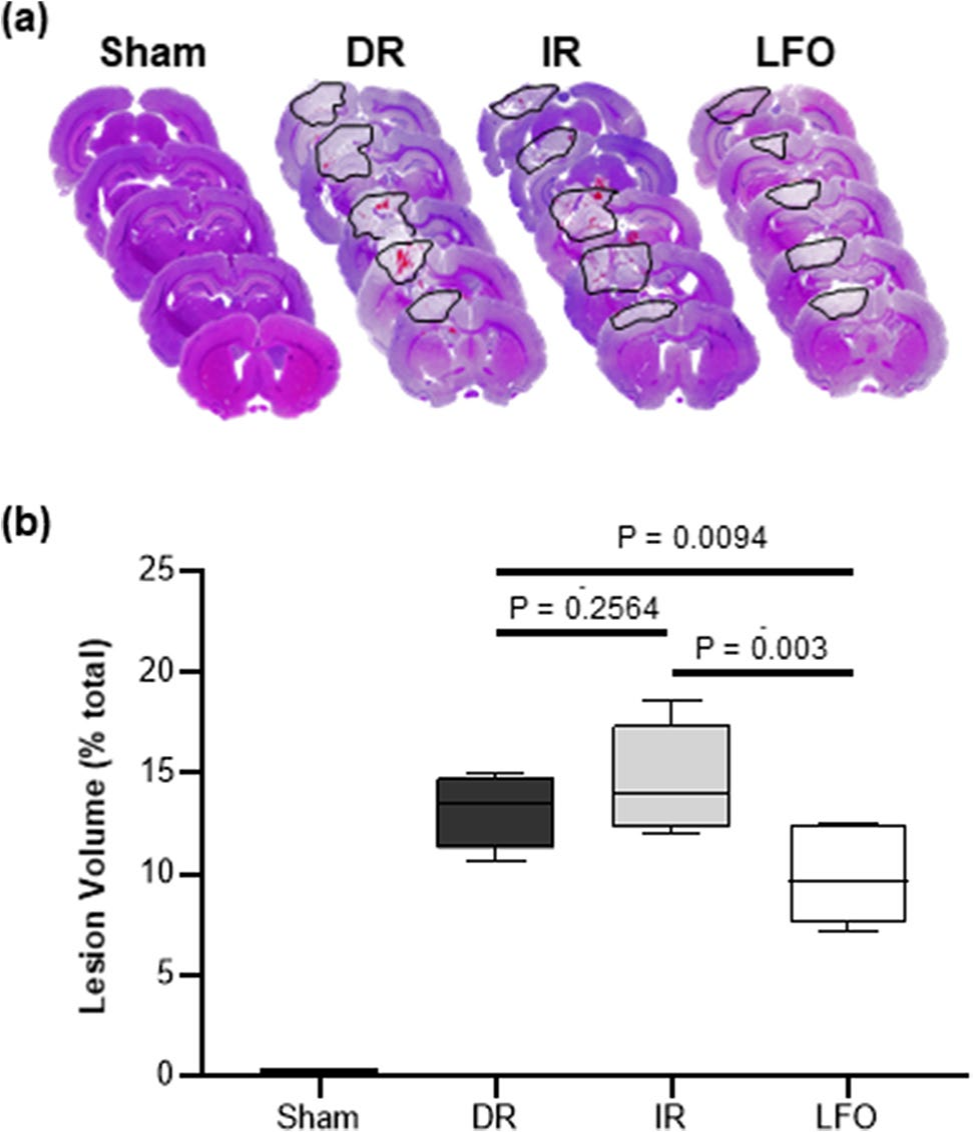
(**a**) Representative photomicrographs of 14 μm thick coronal slices from each experimental group with H&E staining are shown. Lesion regions are circled by black lines. (**b**) TNS-induced low-frequency oscillations in CBF (0.1 Hz) significantly reduced lesion volume at 24 h after TBI + HS. n = 6 ~ 7/group. DR: delayed resuscitation; IR: immediate resuscitation; LFO: low frequency oscillation in CBF.

Lesion volume measurements were used to assess the extent of traumatic injury. Representative images of H&E stained brain coronal sections are shown in Fig. 3A. At 24 h after TBI + HS, animals treated with CBF oscillations at 0.1 Hz demonstrated significantly decreased lesion volumes when compared to DR and IR animals (13.1 ± 1.7% vs. 14.7 ± 2.6% vs. 9.7 ± 2.1%; DR vs. IR vs. LFO; n = 6–7/group) as shown in Fig. 3B. There were no statistically significant differences in DR and IR animals, which represent cerebral hypo- and hyper-perfusion after TBI + HS, respectively. This result indicates that low-frequency oscillations in CBF preserve cell viability in the peri-contusional areas better than the non-oscillatory cerebral hyper-perfusion shown in IR animals.

### Induced low-frequency oscillations in CBF attenuated hypoxic brain injury

**Figure 4:**
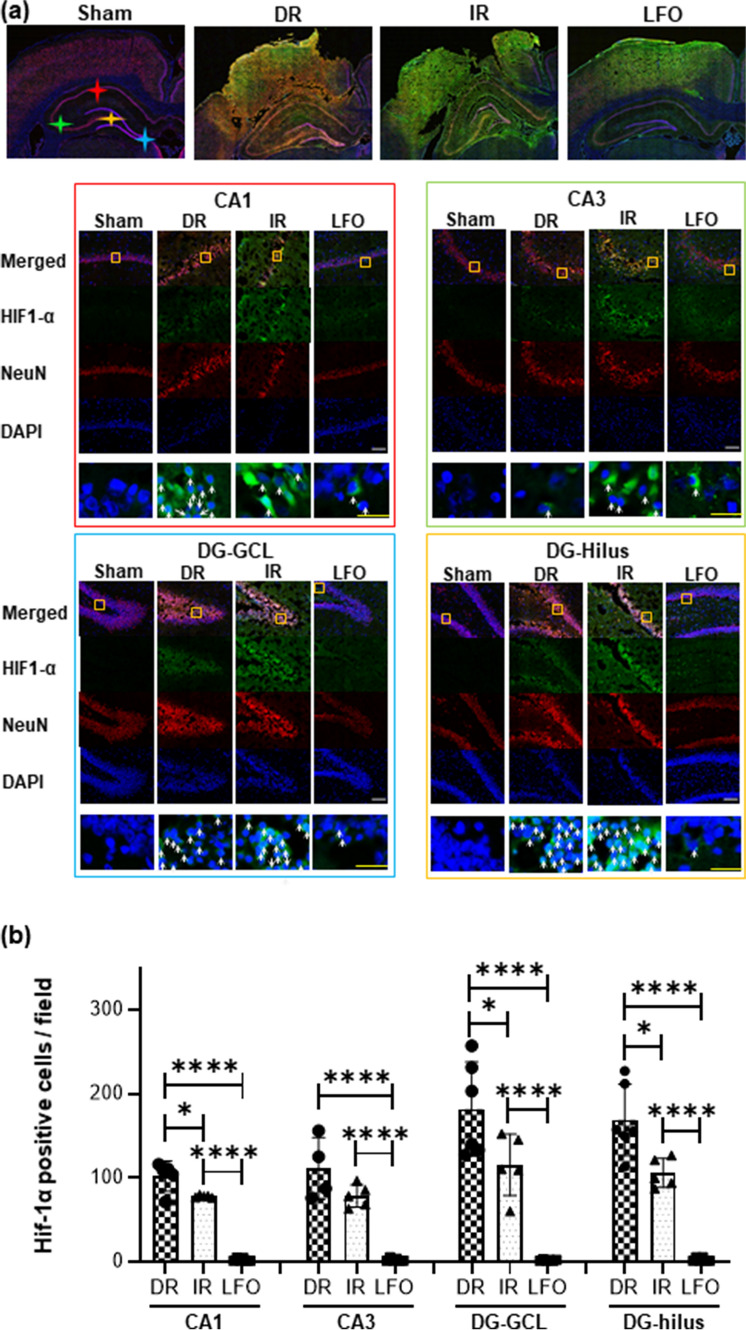
(**a**) Immunofluorescent staining of HIF-1α (green) merged with NeuN (red) and DAPI (blue) in the ipsilateral (injured) hippocampus at 24 h after TBI + HS. Red star: CA1 region; green star: CA3 region; blue star: granular cell layer of dentate gyrus; yellow star: hilus of dentate gyrus. Magnification is 200X, white scale bar = 100 µm, yellow scale bar = 25 µm. (**b**) Quantification of HIF-1α positive cells in the ipsilateral hippocampal subfields of CA1, CA3, and DG. TNS-induced low-frequency oscillations in CBF (0.1 Hz) significantly attenuated hypoxic brain injury. All of the data are presented as mean ± SD. *p < 0.05 versus DR, ^#^*p* < 0.05 versus IR, n = 6–7/group. DR: delayed resuscitation; DG-GCL: dentate gyrus—granule cell layer; IR: immediate resuscitation; LFO: low frequency oscillation in CBF.

Hypoxia-inducible factor-1alpha (HIF-1α) is a sensitive marker of hypoxic stress in neurons^32,33^. The hippocampus is highly vulnerable to hypoxic brain injury^34^. Although it is not often directly damaged by TBI, the hippocampus is subject to the propagation of secondary injury after TBI, and its injury causes long-term memory dysfunction and learning deficits^35^. After TBI + HS, brain sections labeled with HIF-1α and neuronal nuclei (NeuN) demonstrated discrete areas of hypoxia and varying degrees of neuronal disruption (Fig. 4A). The quantification results show that the numbers of neurons expressing HIF-1α was significantly higher in the ipsilateral hippocampal CA1, CA3, and dentate gyrus (DG) both in DR and IR animals compared to LFO animals. The most abundant of HIF-1α positive neurons was in the DG regions of DR and IR animals with a markedly lower number in LFO animals as shown in Fig. 4B. Our findings indicate that cerebral hypoxic injury may be related to microcirculatory derangements given the lack of frank ischemic injury. These changes were mitigated with the administration of low-frequency CBF oscillations, which may boost the diffusion abilities of oxygen to the hypoxic region, restore microcirculatory homeostasis, and prevent reperfusion injury, thus offering new perspectives in the resuscitation of TBI + HS.

### Induced low-frequency oscillations in CBF modules nitric oxide synthase expression

**Figure 5:**
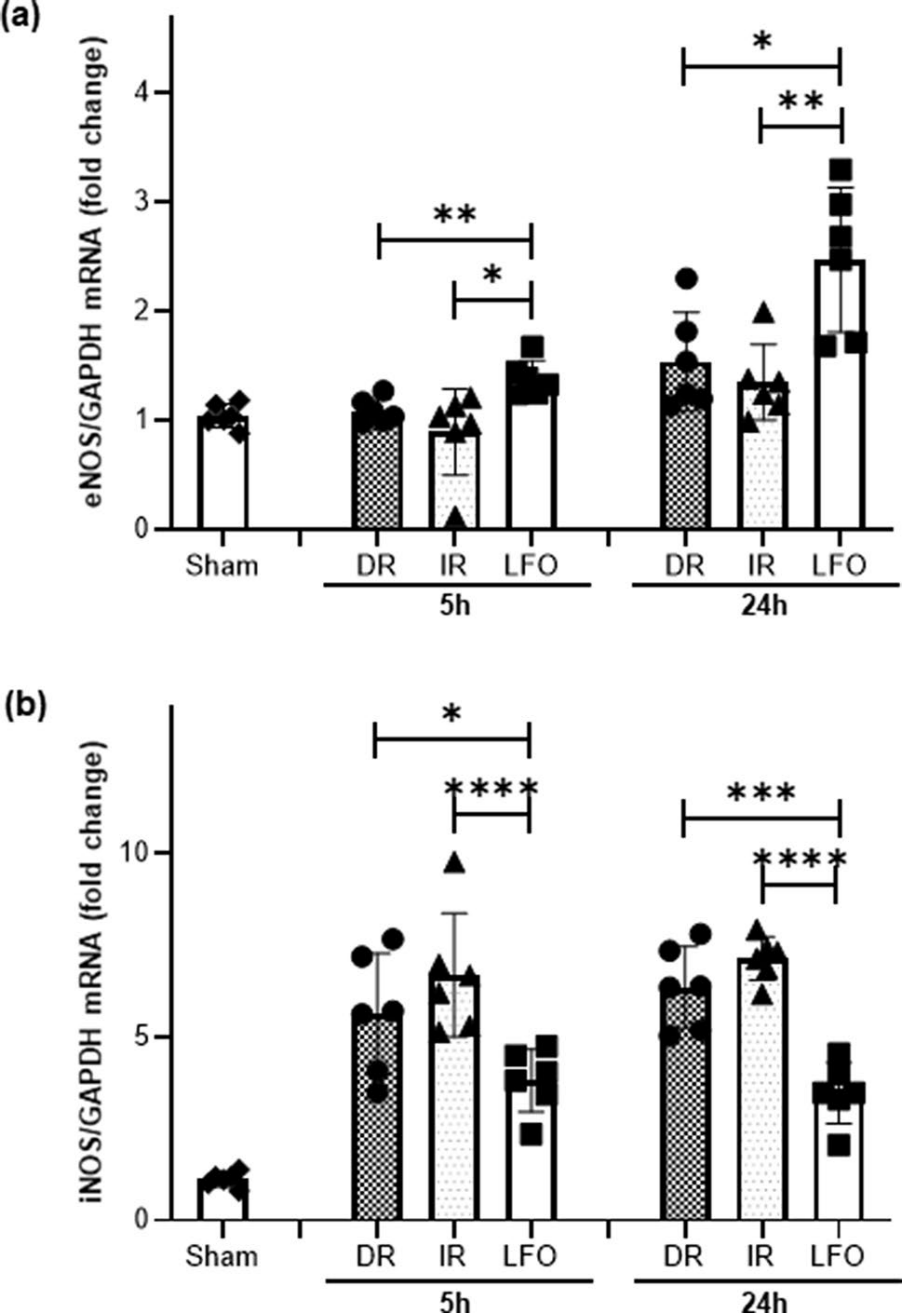
Effect of TNS-induced CBF oscillations at 0.1 Hz on NOS isoforms mRNA expression in peri-contusional brain tissue at 5 h and 24 h after TBI + HS. (**a**) eNOS. (**b**) iNOS. All of the data are presented as mean ± SD, and compared by one-way ANOVA and Student–Newman–Keuls test. n = 5–7/ group. DR: delayed resuscitation; IR: immediate resuscitation; LFO: low frequency oscillation in CBF.

To investigate the effect of TNS-induced CBF oscillations on cerebrovascular endothelium, reverse transcription-quantitative polymerase chain reaction (RT-qPCR) was performed to determine the levels of endothelial nitric oxide synthase (eNOS) and inducible nitric oxide synthase (iNOS). The RT-qPCR assay on the cortex which receives the main trigeminal afferents, showed a significant increase of the expression of the gene coding for eNOS, statistically significant for LFO animals when compared to the IR and DR animals. As shown in Fig. 5A, the mRNA expression levels of eNOS were significantly increased in LFO animals by 28% and 127%, compared with those in the sham group, at 5 h and 24 h after TBI + HS, respectively. In contrast, there were no significant changes both in DR and IR animals compared with those in the sham group. The expression levels of iNOS were significantly increased at the mRNA level in the control, IR, DR, and LFO animals, compared with those in the sham group, both at 5 h and 24 h after TBI + HS as shown in Fig. 5B. However, compared with immediate and delayed resuscitation groups, iNOS was significantly downregulated by TNS-induced CBF oscillations. The results suggest that TNS-induced CBF oscillations at 0.1 Hz protect cerebral endothelium by enhancing eNOS expression and reducing iNOS expression. Further studies are necessary to elucidate the exact mechanism of how TNS-induced CBF oscillations enhance eNOS expression.

### Induced low-frequency oscillations in CBF reduced expression of neuroinflammatory markers

**Figure 6:**
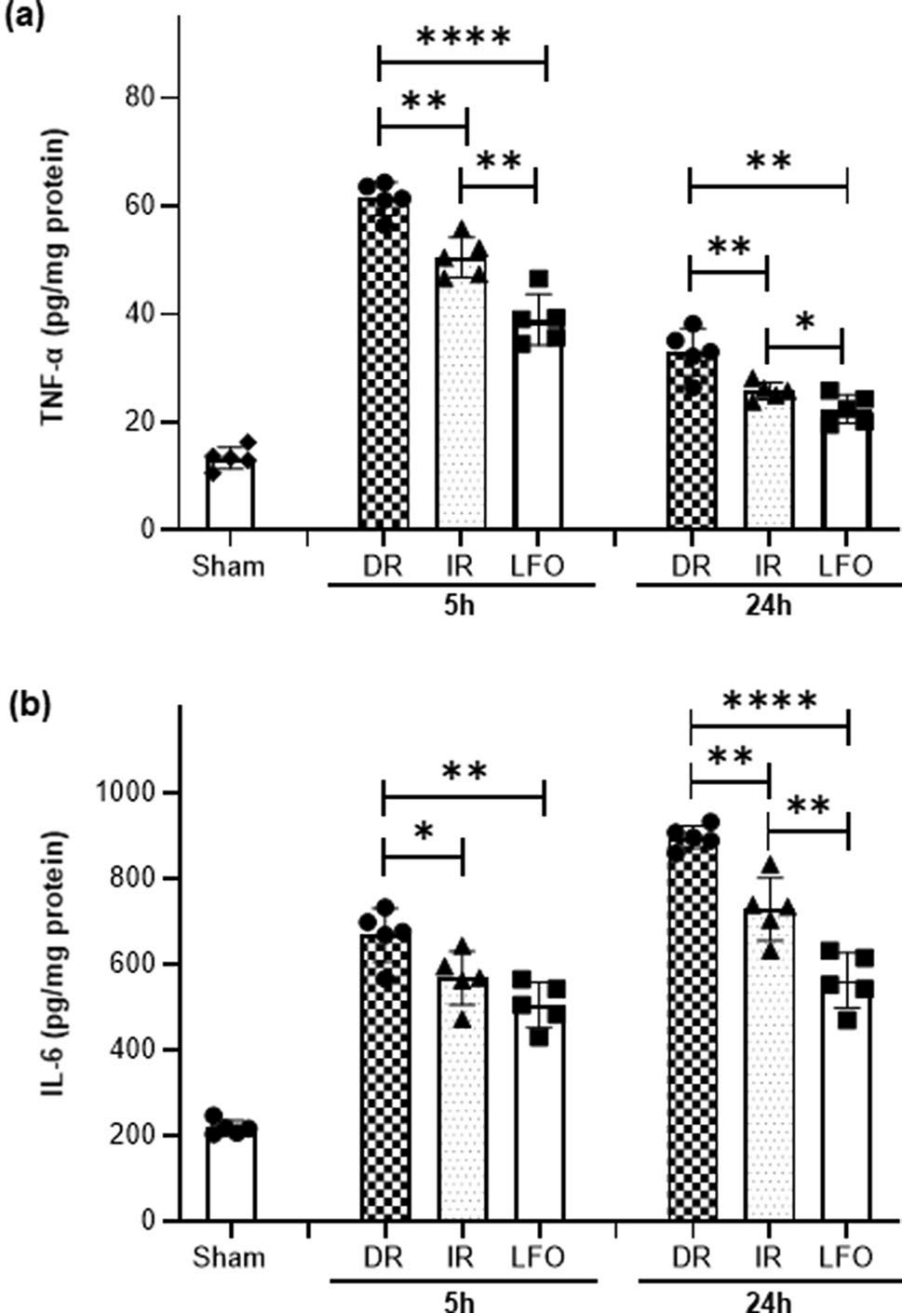
TNS-induced low-frequency oscillations in CBF (0.1 Hz) reduced expression of neuroinflammatory markers. (**a**) Brain tissue levels of TNF-α. (**b**) Brain tissue levels of IL-6. All of the data are presented as mean ± SD, and compared by one-way ANOVA and Student–Newman–Keuls test. n = 5–7/group. DR: delayed resuscitation; IR: immediate resuscitation; LFO: low frequency oscillation in CBF.

TBI + HS initiates a cascade of inflammatory processes that serve to exacerbate the initial injury^36^. As shown in Fig. 6A, brain tissue levels of TNF-α in DR animals increased by 4.6- and 2.5-fold as compared with those in sham-operated animals, at 5 h and 24 h after TBI + HS, respectively. Administration of fluid immediately after TBI + HS (IR) decreased the brain TNF-α levels by 0.8- and 0.5-fold as compared with DR animals, at 5 h and 24 h after TBI + HS, respectively. TNS-induced CBF oscillations significantly decreased brain levels of TNF-α by 0.9- and 0.3-fold as compared with those in IR animals, at 5 h and 24 h after TBI + HS, respectively. Similarly, brain tissue levels of IL-6 after TBI + HS were significantly decreased with the treatment of induced CBF oscillations (Fig. 6B). The results suggest that the TNS-induced CBF oscillations can decrease the neuroinflammatory response more so than immediate fluid resuscitation, implying low-frequency oscillations may play other neuroprotective roles beyond just reducing ischemic/hypoxic injuries.

### Induced low-frequency oscillations in CBF improved neurological recovery

**Figure 7:**
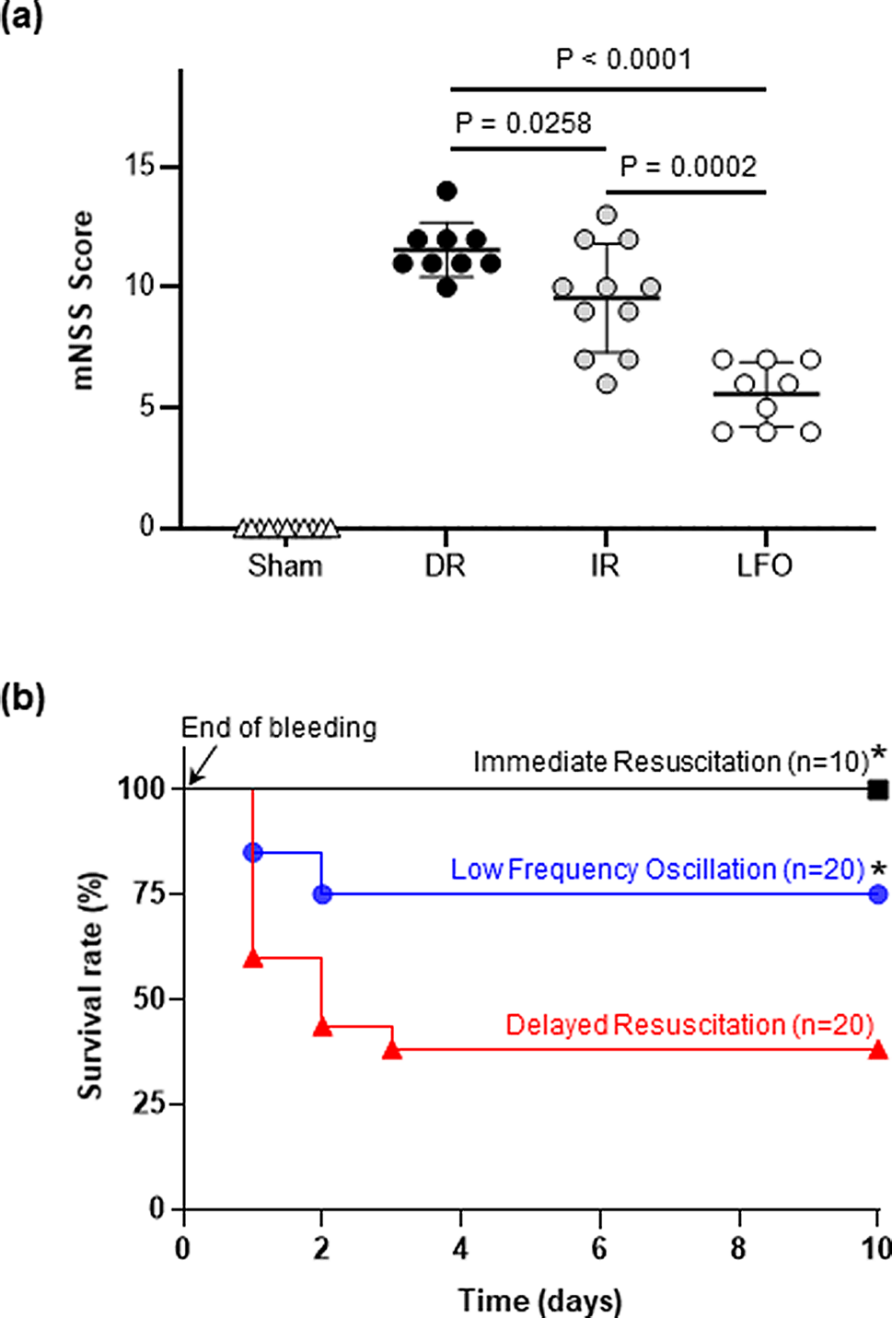
(**a**) TNS-induced low-frequency oscillations in CBF (0.1 Hz) improved neurological recovery at 24 h after TBI + HS. n = 10–16/group. (**b**) Kaplan–Meier survival curves. Induced low-frequency oscillations in CBF (0.1 Hz) improved the 10-day survival rate. *p < 0.05 versus delayed resuscitation group, n = 10 for immediate resuscitation group, n = 20 for delayed resuscitation group, n = 20 for induced low frequency CBF oscillation group.

Functional neurological deficits in animals were evaluated according to the modified Neurological Severity Scale (mNSS) at 24 h after TBI + HS^31^. This includes the sensorimotor function, reflexes and behaviors. As shown in Fig. 7A, mNSS scores increased markedly at 24 h after TBI + HS in DR animals as compared with in sham-operated animals. Although mNSS scores also significantly increased in both IR and LFO animals, the animals treated with the low-frequency CBF oscillations had significantly lower mNSS scores than the animals that received immediate fluid resuscitation. This indicates that maintenance of adequate cerebral perfusion improves neurological function after TBI + HS, however, inducing low-frequency oscillations in CBF plays more important roles to salvage peri-contusional tissue compared to immediate fluid resuscitation.

### Induced low-frequency oscillations in CBF improved the survival rate

A 10-day survival study was performed to assess the impact of induced CBF oscillations via TNS on animal mortality. At 24 h after TBI + HS, the survival rate of DR animals was 60% and further decreased to 40% at 10 days. In contrast, with TNS-induced CBF oscillation, the survival rate increased to 85% and 75%, at 24 h and 10 days, respectively. Immediate fluid resuscitation resulted in a 100% survival. This indicates that the induced low-frequency CBF oscillations confer survival benefits both acutely and also persisting at extended time points up to 10 days following TBI + HS compared to DR animals (Fig. 7B).

## Discussion

TBI is acquired from an external force, possibly resulting in devastating effects to both the cerebrovasculature and neighboring neuronal cells^10^. Disruption of the macroscopic components and microvasculature occurs with the initial primary injury, and often initiates a host of secondary processes that can worsen the impact of the primary injury. The cerebral microcirculation, the main transport and distribution system for oxygen to the cerebral tissue, is significantly compromised in the peri-contusional area after TBI + HS^37^. Despite advances in the understanding of TBI + HS, existing resuscitation approaches inadequately modulate CBF via the microcirculation, and fail to restore normal microcirculatory perfusion leading to hypoxia after TBI + HS as well as may perpetuate reperfusion injury^7,38,39^. Therefore, novel resuscitation strategies that can enhance cerebral microvascular perfusion and, thus, oxygen delivery to tissues in peri-contusional areas are highly desirable. In the present study, we demonstrate in a rat model of TBI + HS that low-frequency CBF oscillations (0.1 Hz) delivered after TBI + HS play a more significant role than improving non-oscillatory cerebral perfusion, by mitigating secondary brain injury, attenuating brain hypoxic injury and neuroinflammation, protecting cerebrovascular endothelium in peri-contusional brain tissue, enhancing neurological recovery, and improving survival rate. To our knowledge, this is the first study where the TNS-induced low-frequency oscillations in CBF have been applied as a therapeutic strategy in TBI + HS.

Spontaneous low-frequency oscillations (0.04–0.15 Hz) in CBF have been observed in individuals with delayed syncope after induced central hypovolemia and are thought to preserve cerebral perfusion^17–20^. While the physiologic mechanisms that govern CBF oscillations remain unclear, studies have shown that oscillatory CBF modulates the release of vasoactive molecules important in maintaining cerebral perfusion via flow-mediated regulatory mechanisms^40^. Specifically, pulsatile blood flow can increase shear stress on the vessel endothelium which stimulates the release of nitric oxide and inhibits endothelin production, thus increasing oxygen delivery to brain tissue^21^. Furthermore, these oscillatory patterns represent an “on–off” feedback mechanism that serves to maintain tissue perfusion in the setting of oxygen deprivation with computational models suggesting that low frequency oscillatory flow is beneficial to tissue oxygenation^15,16^. It might be that brief, rhythmic increases in CBF during the oscillatory stimulus (Fig. 1B,C) provide a pump-like effect leading to improved tissue perfusion via temporary increases in the pressure gradient down the vascular tree. The low-frequency CBF oscillations supply higher amplitudes of localized oxygen delivery relative to oscillations at a higher frequency, therefore enhancing diffusion to the injured mitochondria^16^. This is supported by the findings in this study that animals who received immediate fluid resuscitation had the greatest increase in CBF and overall cerebral perfusion; however, they were found to have brain lesion volumes and HIF-1α expressivity similar to DR animals (cerebral hypo-perfusion), likely due to the failure of fluid resuscitation alone to relieve the effects of the impaired micro-vasculature leading to inadequate microvascular perfusion and ultimately hypoxia after TBI + HS. In contrast, animals undergoing the low-frequency CBF oscillations delivered prior to fluid resuscitation, had significantly attenuated brain hypoxic injury, despite the lack of fluid resuscitation and delayed restoration of perfusion, suggesting an increase in the efficiency and efficacy of oxygen delivery as well as restoration of cerebral microvasculature perfusion.

Microvessel disruption plays a critical role in neuro-functional outcomes after TBI^41,42^. The cerebrovascular endothelium, as a vascular barrier contacting blood directly, is dysfunctional following TBI^43,44^. The endothelial nitric oxide (NO) generated in the cerebrovascular endothelium is one of the most important signaling molecules for CBF autoregulation and there are numerous studies that suggest it is neuroprotective after brain injury^45–47^. Its production mainly depends on endothelial nitric oxide synthase (eNOS) activity, therefore, eNOS activation in the cerebrovascular endothelium plays a significant role in maintaining CBF and oxygenation after TBI, preserving brain microcirculation, inhibiting platelet aggregation, leukocyte adhesion and migration^48,49^. Our results have shown that low-frequency CBF oscillation induced by TNS have upregulated mRNA expression of eNOS by 28% and 127% at 5 h and 24 h after TBI + HS respectively, while there was no significant change in both delayed and immediate resuscitation groups. This suggests that low-frequency CBF oscillations protected cerebral endothelial dysfunction in the peri-contusional areas from further injury that could be partially explained by oscillation-induced increase in vascular wall shear stress that plays a universal role in maintaining the integrity of the endothelium that lines the inner vascular wall^50–52^.

Regulation of CBF is closely related to neurological function^53–55^. Recent studies from our lab^26,30^ and others have demonstrated that TNS can improve cerebral perfusion through the trigemino-cerebrovascular system. In the present study, we demonstrate that TNS-induced CBF oscillations at 0.1 Hz retains the improved CBF achieved after fluid resuscitation, leading to significantly improved neuronal function at 24 h after TBI + HS. The improved CBF that we observed in the animals that had received TNS-induced CBF oscillations is in agreement with previous reports of increased eNOS activity leading to improved CBF after TBI^56–58^. In addition, many reports have shown that iNOS inhibition strategies are neuro-functionally protective possibly by stabilizing macro- as well as microcirculation^59–61^. Our present results show that TNS-induced CBF oscillations also decreased iNOS expression. However, the exact mechanism of how CBF oscillations augment eNOS expression has not been elucidated. Further studies are necessary to explore this.

Besides brain ischemia and hypoxia, TBI + HS also induces inflammatory cascades which are one of the key drivers of worsening neurological outcomes^36^. In this study, we show that brain tissue levels of TNF-α and IL-6 in peri-contusional areas were dramatically increased in both DR and IR animals after TBI + HS. However, the TNS-induced low-frequency CBF oscillation group showed significantly decreased levels of both cytokines, when compared with the animals under similar (DR) or shorter (IR) periods of cerebral ischemia. Generally, it is thought that ischemia triggers the expression of proinflammatory cytokines, subsequently attracting leukocytes into ischemic sites via induction of intercellular adhesion molecule (ICAM)-mediated leukocyte and the adhesion of leukocytes to the luminal wall of microvessels^62,63^. Following adhesion, leukocytes migrate through the vessel wall into the brain parenchyma, triggering a major acute inflammatory response following brain injury^64–68^. We clearly demonstrate that TNS-induced low-frequency CBF oscillations significantly decreased iNOS expression, TNF-α and IL-6 levels, and expression of ICAM1 in peri-contusional tissue (data not shown) despite ischemic conditions as compared to IR animals. These results indicate that the low-frequency CBF oscillations delivered after TBI + HS may not only protect the cerebrovascular endothelium by increasing eNOS expression, but also help to decrease capillary plugging and leukocyte adhesion in the peri-contusional brain tissue, where capillary stasis occurs and exaggerates these phenomena. Our results emphasize the importance of low frequency CBF oscillations beyond simply improving cerebral perfusion and oxygenation after TBI + HS.

Maneuvers such as body tilting and inspiratory resistance breathing have been used to elicit spontaneous CBF oscillations^17–20^. These physiologic maneuvers take advantage of the body’s autonomic reactivity during periods of relative hypotension to enhance the body’s tolerance to hypotension; however, the physiologic response can be highly variable and inconsistent between individuals^69,70^. In other words, not all subjects have the same degree of spontaneous CBF oscillations and therefore are not equally capable of tolerating hypotensive injury. Furthermore, these approaches would be difficult to apply to patients with TBI + HS. To overcome this, we demonstrate here that specific calibration of TNS parameters can consistently generate low-frequency oscillations in CBF, as seen in subjects that exhibit high tolerance to central hypovolemia. TNS is well-suited to induce CBF oscillations due to its rich network supplying a large portion of extrinsic neural supply to the blood vessels of the brain as well as its connections to various brainstem regions known to intrinsically modulate the cerebral microvasculature^22,25,26^. Proximal to the Virchow-Robin space, the trigeminal nervous network, innervates the much of the cerebral vasculature^71–73^. The results described herein show that TNS not only generated the controllable CBF oscillations in the targeted low frequency range, but also gradually improved the overall CBF amplitude. Given this evidence, it is reasonable to apply TNS as a tool to induce low-frequency CBF oscillations as a treatment strategy for TBI + HS.

This study has some limitations. First, animals were maintained under general anesthesia throughout the duration of the experiment. It is known that inhaled anesthetics, isoflurane in particular, exhibit neuroprotective properties that can confound our results^74^. Additionally, there is evidence to suggest that isoflurane augments eNOS protein expression in animals^75^. Furthermore, most clinical investigations of the physiology of spontaneous CBF oscillations have been performed in conscious subjects^17–20^. While these limitations were minimized by sham, delayed and immediate resuscitation experimental groups, the low frequency oscillation treatment of awake animals may represent another avenue of research to limit the effects of general anesthesia. Second, it should be noted that we proved our hypothesis that induced low-frequency CBF oscillations alleviate impaired cerebral microcirculation in peri-contusional brain tissue by using indirect measurements such as lesion volume, HIF-1α and eNOS expression, neuroinflammation, etc. It would have been ideal if the cerebral microcirculation were assessed directly using a capillary anemometer^76^ or in vivo two-photon laser scanning microscopy^77^ over the peri-contusional cortex. Third, most findings discussed herein represent outcomes at 5 h and 24 h after TBI + HS. However, long-term outcomes significantly influence behavior and higher cognitive function and have significant impact in the recovery from trauma^78,79^. Future work should investigate whether the neuroprotective effects of induced CBF oscillations persist long-term as well. Fourth, only male rats of the similar age (9–11 weeks) were studied. The outcomes from male and female rats should be compared to investigate the potential sex and age differences of the treatment for TBI outcomes^80,81^. Finally, we have only studied outcomes with CBF oscillations at 0.1 Hz. In the future, we plan to study the effect of CBF oscillations at different frequencies.

In conclusion, our results demonstrate that TNS-induced low-frequency CBF oscillations at 0.1 Hz play a more significant role in preserving peri-contusional brain tissue than improving non-oscillatory cerebral perfusion or volume expansion in an animal model of severe TBI + HS. Our findings provide novel insights into the neuroprotective strategies employing low-frequency oscillations in CBF in injured brains to improve cerebral microcirculation and oxygenation. Although the experimental results shown in this study are promising, further studies are needed to understand the effects of CBF oscillations with different frequencies and to evaluate their effects on other organs.

## Data Availability

The datasets generated during and/or analysed during the current study are available from the corresponding author on reasonable request.
